# Calcified Thrombus in Right Atrium: Rare but Treatable Complication of Long-term Indwelling Central Venous Catheter

**DOI:** 10.4021/cr24w

**Published:** 2011-07-25

**Authors:** Marianna Fabi, Valentina Gesuete, Gabriella Testa, Anna Balducci, Fernando Maria Picchio, Gaetano Gargiulo

**Affiliations:** a Pediatric Cardiology Unit, S.Orsola-Malpighi Hospital, Bologna, Italy; b Pediatric Cardiac Surgery Unit, S.Orsola-Malpighi Hospital, Bologna, Italy

**Keywords:** Catheter-related central thrombosis, Calcified thrombus, Complication, Surgical removal

## Abstract

Catheter-related central thrombosis is a rare complication of long-term central line. We describe the case of an asymptomatic boy who was diagnosed a calcified thrombus in right atrium eight years after the removal of a long-term central venous device. Although the most appropriate therapeutic approach for managing floating right heart thrombi remains to be determined, surgical removal is an effective and safe procedure for calcified long-standing thrombus and it is to be preferred in elective conditions especially in young asymptomatic patients without hemodynamic involvement, that are at low risk of surgery-related morbidity and mortality.

## Introduction

Catheter-related central thrombosis is a rare complication of long-term central line [1, 2] especially in patients unexposed to parenteral nutrition. Children treated for acute lymphoblastic leukaemia (ALL) are at major risk of thromboembolic events occurring during or after chemotherapy [3], because of indwelling venous device [4], and because some agents, as asparaginase, reduce plasma concentration of most coagulation proteins, antithrombin levels included [5]­. Right atrial thrombus can have rare but dramatic complications [6, 7] because it can embolize causing pulmonary embolism or paradoxical systemic embolism. Management of this condition remains challenging [8] especially in asymptomatic patients with old-thrombus.

We report the case of an asymptomatic boy who was diagnosed a thrombus in right atrium by echocardiogram eight years after the removal of a long-term central venous device used for chemotherapy administration.

## Case Report

**Figure 1:**
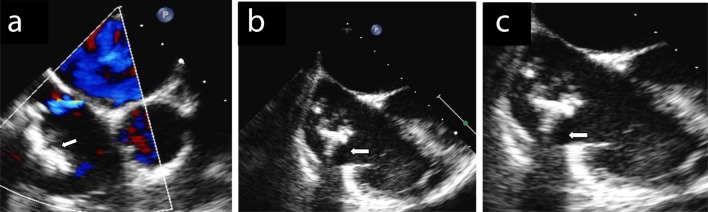
(a, b, c) Transesophageal echo confirmed the location and the extent of the lesion and showed a large patent foramen ovalis through which a left to right shunt was detected after infusion of contrast media in basal conditions and right to left shunt after Valsalva maneuver.

**Figure 2:**
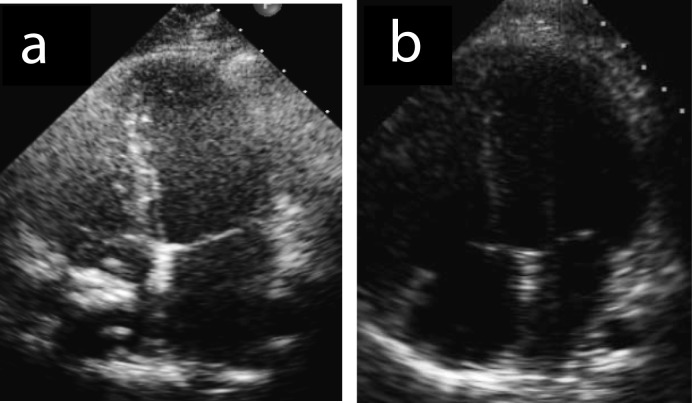
(a, b) Echocardiogramm post-surgery revealed absence of thrombus.

A 14-year-old boy was referred to our service to get a cardiologic evaluation with electrocardiogram (ECG) and cardiac ultrasonography as part of pre-partecipation screening to play rugby competitions. He was clinically fine, medical examination was unremarkable, and ECG was normal. Transthoracic echocardiography (TTE) evidenced a hyperechogenic polyglobulated calcified mass, suggesting it was an old formation, sizing 22 x 11 mm, consistent with an organised thrombus, with a thin pedicule of implantation in the floor of the right atrium, close to ostium of inferior cava vein, floating in atrium, without obstruction of systemic venous flow or tricuspidalic valve. He had never been symptomatic for embolic events and he had been performing sport activity (rugby) without any limitation for six months. The medical history included an ALL diagnosed when he was 6 years old. At that time a long-term indwelling central line (Broviac) via internal left jugular vein was placed without difficulty for chemotherapy administration. He was treated with Protocol AIEOP ALL 2000 that includes asparaginase. For standard evaluation of anthracycline toxicity at the beginning of chemotherapy he had a routine two-dimensional echocardiogram that did not show anomaly. During the treatment he had not bacterial nor mycotic sepsis and the Broviac was never used for parenteral nutrition. The catheter was removed uneventfully six months after completion of chemotherapy, thus eighteen months after implantation. To better define the characteristics of the mass and of interatrial septum, we performed a transesophageal echo (TEE) that confirmed the location and the extent of the lesion and showed a large patent foramen ovalis (PFO) through which a left to right shunt was detected after infusion of contrast media in basal conditions and right to left shunt after Valsalva manouver (Fig. 1a, b, c). Transcranic Doppler (TCD) was positive for moderate shunt. We excluded other diagnosis such as congenital structures (Chiari network, redundant Thebesian or Eustachian valve, aneurysm of atrial septum) or acquired disease (vegetations and tumors). Patient was not eligibile for intravenous thrombolysis because the formation was considered old. To prevent embolic accidents, especially cerebral lesions, the boy underwent thrombectomy via sternotomy and closure of PFO. Macroscopically the lesion was serpiginous and polylobulated with irregular profile, calcified and very fragile, easily fragmentable. The formation was successfully removed without complications (Fig. 2a, b). Hospital stay lasted seven days. Seven months after surgical intervention, he was allowed to play sport thanks to complete resolution of disease.

## Discussion

Mobile right atrial thrombi are a rare but potentially catastrophic phenomenon, because they can embolize at any moment requiring emergency treatment. Immobile thrombi can develop in situ favoured by blood stagnation and usually they have a good prognosis when treated with heparin. Right atrial thrombus is a known complication of long-term central venous catheter (CVC), and even if uncommon in children [9-12], it is not a rare finding if routinely screened in children with cancer [13]. The incidence of cardiac thrombus increases when used for total parenteral nutrition, due to hyperosmolarity, and in patients treated for tumors, due to hypercoagulability state subsequent to some chemotherapeutic agents, as asparaginase, which decrease plasma levels of proteins, included coagulation proteins [5, 14-17]. Moreover, these patients are at major risk for mycotic or bacterial sepsis and prolonged statement of central line; endothelial injury due to the tip of CVC can predispose and trigger the development of thrombus. Further, it seems that tip position in right atrium is associated with a higher risk of developing thrombus than when it is located in superior vein cava or in its junction with right atrium [18]. In our patient the first echographic control after the placement was normal; catheter was used just for infusion of chemotherapic agents and removed six months after the completion of therapy, that is eighteen months after the implantation, and it never had problems when in place. Cardiac ultrasonography represents a valid and rapid tool to monitor catheter and detects potentially life-threatening but treatable formations [19]: TTE can easily and quickly diagnose intracardiac mass and TEE can add further details to describe it, and define interatrial septum and shunts through PFO. TCD was useful to prove paradoxical embolus, completing US study. Differential diagnosis must include congenital and acquired conditions. Floating right atrial thrombus can cross interatrial septum via PFO or embolize in pulmonary vascular bed with dramatic consequences, so, even if it was likely to be a clinically silent old formation, we decided for cardiac surgery because the patient was not eligible for thrombolysis or anticoagulation. In this case a low risk operation, such as closure of PFO and thrombus removal, allowed to ensure this otherwise healthy teen-age boy a normal quality of life, performing any kind of physical activity.

We emphasize the need for removing CVC as soon as possible after completion of treatment and for monitoring long-term indwelling central line especially in patients receiving chemotherapy and parenteral nutrition even in asymptomatic children in absence of signs of malfunctions, in order to promptly identify thrombus formation. Treatment for this condition is not standardized yet (thrombolysis, anticoagulation, surgical thrombectomy) [20] and the choice depends on physician’s judgement. We think that most appropriate strategy should be individualised: in our case surgical option was indicated to completely treat the high risk thrombus in presence of PFO in an asymptomatic young boy wishing a normal quality of life performing all the activities as a teen age healthy boy should do. Elective surgery is a low risk and completely effective procedure.

The presence of intracardiac thrombi should be monitored in children with long-term central line for chemotherapy, and echocardiography is a quick and valid instrument to detect this life-threating but treatable condition. Although the most appropriate therapeutic approach for managing floating right-heart thrombi remains to be determined (thrombolysis, anticoagulation, cardiac surgery), we suggest that surgical removal is an effective and safe procedure for calcified thrombus and it is to be preferred in elective conditions especially in young asymptomatic patients without hemodynamic involvement, who are at low risk of surgery-related morbidity and mortality.
